# Validation of human immunodeficiency virus diagnosis codes among women enrollees of a U.S. health plan

**DOI:** 10.1186/s12913-024-10685-x

**Published:** 2024-02-22

**Authors:** Gaia Pocobelli, Malia Oliver, Ladia Albertson-Junkans, Gabrielle Gundersen, Aruna Kamineni

**Affiliations:** Kaiser Permanente Washington Health Research Institute, 1730 Minor Avenue, Suite 1600, 98101 Seattle, Washington, USA

**Keywords:** HIV, Predictive value of tests, Validation study, Electronic health records, ICD codes

## Abstract

**Background:**

Efficiently identifying patients with human immunodeficiency virus (HIV) using administrative health care data (e.g., claims) can facilitate research on their quality of care and health outcomes. No prior study has validated the use of only ICD-10-CM HIV diagnosis codes to identify patients with HIV.

**Methods:**

We validated HIV diagnosis codes among women enrolled in a large U.S. integrated health care system during 2010–2020. We examined HIV diagnosis code-based algorithms that varied by type, frequency, and timing of the codes in patients’ claims data. We calculated the positive predictive values (PPVs) and 95% confidence intervals (CIs) of the algorithms using a medical record-confirmed diagnosis of HIV as the gold standard.

**Results:**

A total of 272 women with ≥ 1 HIV diagnosis code in the administrative claims data were identified and medical records were reviewed for all 272 women. The PPV of an algorithm classifying women as having HIV as of the first HIV diagnosis code during the observation period was 80.5% (95% CI: 75.4–84.8%), and it was 93.9% (95% CI: 90.0-96.3%) as of the second. Little additional increase in PPV was observed when a third code was required. The PPV of an algorithm based on ICD-10-CM-era codes was similar to one based on ICD-9-CM-era codes.

**Conclusion:**

If the accuracy measure of greatest interest is PPV, our findings suggest that use of ≥ 2 HIV diagnosis codes to identify patients with HIV may perform well. However, health care coding practices may vary across settings, which may impact generalizability of our results.

## Background

Research on the quality of health care received by persons with human immunodeficiency virus (HIV), and their health outcomes, can be facilitated by efficiently identifying cohorts of patients with HIV using electronic health record (EHR) data (e.g., claims) [1, 2]. Algorithms using EHR data have been developed and report good accuracy for identifying a cohort with HIV, however, these algorithms require use of not only diagnosis codes for HIV, but also laboratory and/or medication data [1, 3, 4]. Although supplementing diagnosis codes with laboratory and medication data may improve algorithm performance, laboratory and medication data may not be available from a given EHR data source [5]. The applicability of those algorithms will be limited to settings where those various data sources are available.

Few studies have reported the accuracy of algorithms using only diagnosis codes for HIV [1, 6], and to our knowledge, no prior study has reported the accuracy of using HIV diagnosis codes from the International Classification of Diseases Tenth Revision, Clinical Modification (ICD-10-CM) era (i.e., October 1, 2015 and later in the U.S.) only [7]. Although most HIV diagnosis codes are equivalent between International Classification of Diseases Ninth Revision, Clinical Modification (ICD-9-CM) [8] and ICD-10-CM, there is a notable difference with the addition in ICD-10-CM of codes specific to HIV in pregnancy, childbirth, and the puerperium (O98.711-O98.73). No such code group exists in ICD-9-CM.

Using data from a large cohort study of women enrolled in a U.S. integrated health care delivery system that spanned ICD-9-CM and ICD-10-CM eras, we sought to validate various claims-based algorithms that differed according to the type, frequency, and timing of the HIV diagnosis codes. As it is guideline-recommended that CD4 cell counts be measured regularly in patients with HIV [9], we also examined algorithms that additionally included procedure codes for CD4 testing.

## Methods

### Study population

This study was approved by the Kaiser Permanente Washington (KPWA) institutional review board and they issued a waiver of informed consent to collect patient health record data. All methods were performed in accordance with relevant guidelines and regulations. The setting for this validation study was KPWA’s integrated health care delivery system in Washington state. The base population included KPWA members of a multisite cohort study designed to evaluate the cervical cancer screening process, part of the National Cancer Institute-funded Population-based Research to Optimize the Screening Process (PROSPR II) consortium [10]. KPWA PROSPR II cohort members were women enrolled in KPWA or Molina Healthcare (i.e., covered by Medicaid) who were 18–89 years of age during 2010–2020; had a selected, assigned, or attributed KPWA primary care provider; and were residents of the catchment area of the Seattle-Puget Sound Surveillance Epidemiology and End Results (SEER) registry (*N* = 456,461 women). Cohort follow-up time accrued until the earliest occurrence of the following: a > 90-day gap in KPWA enrollment or a > 90-day gap in having a selected, assigned or attributed KPWA primary care provider (64.7%); a > 90-day gap in residency in the Seattle-Puget Sound SEER registry catchment area (1.9%); age ≥ 90 years (1.3%); death (2.5%); or December 31, 2020 (29.6%). Cohort members were not permitted to re-enter the cohort. For the present analysis, we identified all PROSPR II cohort members who had ≥ 1 ICD-9-CM or ICD-10-CM diagnosis code for HIV in the KPWA administrative claims data during cohort follow-up (*N* = 272 women). KPWA administrative claims data include health care claims for diagnoses and procedures received by KPWA enrollees in inpatient and outpatient settings. All HIV diagnosis codes are listed in Table 1, items #4 and #5.

**Table 1:** Characteristics of women with ≥ 1 HIV diagnosis code during 2010–2020 at Kaiser Permanente Washington

Patient Characteristic	*N* = 272 n (%)
Age at first HIV diagnosis code, years	
Mean (standard deviation)	41.2 (13.1)
Median (interquartile range)	40 (32–49)
18–29	45 (16.5)
30–39	89 (32.7)
40–49	77 (28.3)
50–59	37 (13.6)
≥60–89	24 (8.8)
Race/ethnicity^a,b^	
Hispanic	24 (10.4)
Native American/Alaska Native	0 (0.0)
Non-Hispanic Black	98 (42.6)
Non-Hispanic White	89 (38.7)
Multiple Races/Other Race	19 (8.3)
Unknown	42
Insurance at the time of the first HIV diagnosis code	
Medicaid	15 (5.5)
Medicare	21 (7.7)
Commercial/private payer	236 (86.8)
Year of first HIV diagnosis code	
2010–2014	129 (47.4)
2015–2020	143 (52.6)
Duration of follow-up, years^c^	
Mean (standard deviation)	3.1 (3.0)
Median (interquartile range)	2.0 (0.9–3.9)
0 to < 1 year	115 (27.2)
1 to < 3 years	105 (38.6)
3 to < 5 years	40 (14.7)
≥5 years	53 (19.5)

^a^Percentages calculated after excluding women with unknown race/ethnicity

^b^Other race includes non-Hispanic Asian and non-Hispanic Native Hawaiian/other Pacific Islander

^c^Number of years from the first HIV diagnosis code during cohort follow-up through cohort exit date. The distribution of cohort exit reasons was as follows: disenrolled from KPWA or no longer had a KPWA primary care provider (*n* = 144; 52.9%); end of study period (December 31, 2020) (*n* = 112; 41.2%); died (*n* = 10; 3.7%); moved out of the Puget Sound SEER registry catchment area or attained 90 years of age (*n* = 6; 2.2%)

### Confirmation of HIV diagnosis

Among the *N* = 272 women with ≥ 1 day with an HIV diagnosis code during cohort follow-up, we sought to ascertain the earliest date during cohort follow-up when an HIV diagnosis was confirmed in the medical record. To do so, we first identified the date of each occurrence of an HIV diagnosis code in the administrative claims data during cohort follow-up. Medical records were reviewed by trained medical record abstractors during a +/- 6-month window from each patient’s HIV diagnosis code to ascertain the gold standard definition of an HIV diagnosis (defined below). Reviews were conducted in chronologic order and once the gold standard definition of HIV was confirmed, no review of subsequent codes was conducted. The gold standard definition of an HIV diagnosis was a medical record-abstracted clinician’s note stating that the patient had a diagnosis of HIV, or in the absence of a clinician’s note, laboratory evidence in the medical record of an HIV diagnosis (i.e., a positive result from an HIV viral load test [any detectable viral load threshold] or HIV antibody tests). Patients were considered to have a confirmed HIV diagnosis as of the earliest validated HIV diagnosis code during cohort follow-up.

### Statistical analysis

We described the study cohort according to demographic and clinical characteristics. We calculated the positive predictive values (PPV) and Wilson 95% confidence intervals (CIs) [11] for various algorithms defined by HIV diagnosis and CD4 procedure codes [12] present in the claims data. For each algorithm, the denominator of the PPV included all women who met the algorithm criteria during cohort follow-up. The numerator of the PPV included all women with a confirmed HIV diagnosis on or before the date the algorithm criteria were met. For example, the PPV of an algorithm requiring ≥ 2 days with an HIV diagnosis code was calculated as the percent of women with ≥ 2 days with an HIV diagnosis code in their claims data during cohort follow-up who had a medical chart confirmed-HIV diagnosis as of the second code.

We evaluated the PPV of algorithms that varied by time period examined (i.e., the ICD-9-CM era [the time period when only ICD-9-CM codes were in use at KPWA] and the ICD-10-CM era [the time period when only ICD-10 codes were in use at KPWA]). When examining the PPV of HIV diagnosis codes during the ICD-10-CM era (i.e., as of 10/1/2015 at KPWA), we only included individuals whose first HIV diagnosis code during cohort follow-up occurred during 10/1/2015-12/31/2020.

All analyses were conducted in Stata 17.

## Results

Among the 272 cohort members with ≥ 1 day with an HIV diagnosis code in the administrative claims data during cohort follow-up (2010–2020), the medical record was identified and reviewed for all 272 women. Demographic and clinical characteristics at the time of the first HIV diagnosis code are described in Table 2. For age, 49.2% of women were < 40 years, 41.9% were 40–59 years, and 8.8% were 60–89 years. Among the 230 women with known race or Hispanic ethnicity information (84.6% of the 272 women), 42.6% were non-Hispanic Black, 38.7% non-Hispanic white, 10.4% Hispanic, and 8.3% multiple races/other race. The preponderance of women (86.8%) had commercial or private payer insurance, 7.7% were covered by Medicare, and 5.5% were covered by Medicaid. In slightly less than half of women, the first HIV diagnosis code during cohort follow-up occurred during 2010–2014, and in the remaining women it occurred during 2015–2020. The median duration of follow-up from the first HIV code through cohort exit was 2.0 years (interquartile range: 0.9–3.9 years).

**Table 2:** Positive predictive values of health care claims-based algorithms for identifying patients with HIV, Kaiser Permanente Washington (2010–2020), *N* = 272 women

Algorithm description	Number of women meeting algorithm criteria (N)	Number of women with confirmed HIV as of the date the algorithm criteria were met (n)	Positive predictive value ([n/N]*100) (95% CI)
1. Number of days with an HIV diagnosis code			
a) ≥ 1 day with an HIV diagnosis code	272	219	80.5 (75.4–84.8)
b) ≥ 2 days with an HIV diagnosis code	230	216	93.9 (90.0-96.3)
c) ≥ 3 days with an HIV diagnosis code	219	213	97.2 (94.2–98.7)
2. ICD-9-CM era^b,c^			
a) ≥ 1 day with an HIV diagnosis code during 1/1/2010-9/30/2015 (ICD-9-CM era)	139	108	77.7 (70.1–83.8)
b) ≥ 1 day with an HIV diagnosis code during 1/1/2010-12/31/2012 (earlier ICD-9-CM era)	106	83	78.3 (69.5–85.1)
c) ≥ 1 day with an HIV diagnosis code during 1/1/2013-9/30/2015 (later ICD-9-CM era)	33	25	75.7 (59.0-87.2)
3. ICD-10-CM era^b,d^			
a) ≥ 1 day with an HIV diagnosis code during 10/1/2015-12/31/2020 (ICD-10-CM era)	133	110	82.7 (75.4–88.2)
b) ≥ 1 day with an HIV diagnosis code during 10/1/2015-12/31/2017 (earlier ICD-10-CM era)	38	27	71.1 (55.2–83.0)
c) ≥ 1 day with an HIV diagnosis code during 1/1/2018-12/31/2020 (later ICD-10-CM era)	95	83	87.4 (79.2–92.6)
4. Individual ICD-9-CM HIV diagnosis codes^b^			
a) ≥ 1 day with ICD-9-CM HIV diagnosis code, 042 (“HIV disease”)	110	91	82.7 (74.6–88.7)
b) ≥ 1 day with ICD-9-CM HIV diagnosis code, V08 (“Asymptomatic HIV infection status”)	98	82	83.7 (75.1–89.7)
c) ≥ 1 day with an ICD-9-CM HIV diagnosis code, 079.53 (“Infection, conditions classified elsewhere & unspecified; HIV, type 2”)	2	2	100.0 (34.2–100.0)
d) ≥ 1 day with ICD-9-CM diagnosis code 795.71 (“Nonspecific serological evidence of HIV”)	5	2	40.0 (11.8–76.9)
5. Individual ICD-10-CM HIV diagnosis codes^b^			
a) ≥ 1 day with ICD-10-CM diagnosis code B20 (“HIV disease”)	119	104	87.4 (80.2–92.2)
b) ≥ 1 day with ICD-10-CM diagnosis code Z21 (“Asymptomatic HIV infection status; HIV positive NOS”)	100	92	92.0 (85.0-95.9)
c) ≥ 1 day with ICD-10-CM diagnosis code B97.35 (“HIV, type 2 as the cause of diseases classified elsewhere”)	0	0	N/A
d) ≥ 1 day with ICD-10-CM diagnosis code O98.711-O98.73 (“HIV disease complicating pregnancy, childbirth and the puerperium”)	10	10	100.0 (72.2–100.0)
6. HIV diagnosis codes and CD4 procedure codes^a^			
a) ≥ 1 day with an HIV diagnosis code & ≥1 day with a CD4 procedure code on or after the day of the first HIV code.	218	205	94.0 (90.1–96.5)

HIV: human immunodeficiency virus; CI: confidence interval; ICD-9-CM: International Classification of Diseases Ninth Revision, Clinical Modification; ICD-10-CM: International Classification of Diseases Tenth Revision, Clinical Modification

^a^CD4 procedure codes used were Current Procedural Terminology (CPT) 86,360 (“T cells; absolute CD4 and CD8 count, including ratio”) and 86,361 (“T cells; absolute CD4 count”)

^b^Each row includes only those women whose first HIV diagnosis code during cohort follow-up occurred on or after the start of the time period examined. For individual ICD-9-CM codes (item #4) the time period examined was 1/1/2010-9/30/2015, and for individual ICD-10-CM codes (item #5) it was 10/1/2015-12/31/2020

^c^The ICD-9-CM era corresponds to time during the study period when ICD-9-CM codes were in use at KPWA, i.e., 1/1/2010-9/30/2015

^d^The ICD-10-CM era corresponds to the time during the study period when ICD-10-CM codes were in use at KWPA, i.e., 10/1/2015-12/31/2020

Among the 272 women with ≥ 1 day with an HIV diagnosis code in the administrative data during cohort follow-up, a total of 227 women met the gold standard definition of an HIV diagnosis at some point during cohort follow-up (data not shown). Of these 227 women, the HIV diagnosis was confirmed via a provider’s note stating the patient had HIV in 224 women (98.7%), and in the remaining 3 women (1.3%), HIV was confirmed via laboratory evidence alone.

Of the 272 women with ≥ 1 day with an HIV diagnosis code in the administrative data during cohort follow-up, HIV was confirmed as of the first day with an HIV diagnosis code in 219 women (PPV = 80.5%; 95% CI: 75.4–84.8%; Table 1). The PPV of the algorithm requiring ≥ 2 days with an HIV diagnosis code was greater (PPV = 93.9%; 95% CI: 90.0-96.3%). The PPV point estimate increased slightly when ≥ 3 days with an HIV diagnosis code was required (PPV = 97.2%; 95% CI: 94.2–98.7%) but the 95% CI overlapped with the estimate that required ≥ 2 days with an HIV diagnosis code.

Similar PPVs were observed for ICD-9-CM and ICD-10-CM HIV diagnosis code-based algorithms, 77.7% (95% CI: 70.1–83.8%) and 82.7% (95% CI: 75.4–88.2%), respectively (Table 1).

We additionally examined individual codes within each era. The PPVs were similar across the two most commonly observed ICD-9-CM codes: ≥1 day with a V08 code (“Asymptomatic HIV infection status”) was 83.7% (95% CI: 75.1–89.7) and ≥ 1 day with a 042 code (“HIV disease”) was 82.7 (95% CI: 74.6–88.7) (Table 1). Only a handful of women had the two remaining ICD-9-CM codes (079.53 [*n* = 2 women] and 795.71 [*n* = 5 women]) and PPV estimates were imprecise.

The PPVs were also similar across the two most commonly observed ICD-10-CM codes: ≥1 day with a Z21 code (“Asymptomatic HIV infection status; HIV positive NOS”) was 92.0% (95% CI: 85.0-95.9) and ≥ 1 day with a B20 code (“HIV disease”) was 87.4% (95% CI: 80.2–92.2) (Table 1). The remaining ICD-10-CM code group was the new group added to ICD-10-CM, O98.711-O98.73 (“HIV disease complicating pregnancy, childbirth and the puerperium”); the PPV of ≥ 1 day with O98.711-O98.73 was 100.0% (95% CI: 77.2–100.0).

We also examined the PPV of an algorithm requiring ≥ 1 day with an HIV diagnosis code and ≥ 1 day with a CD4 procedure code on or after the first day with an HIV code and the PPV was 94.0% (95% CI: 90.1–96.5; Table 1).

## Discussion

The use of diagnosis codes to identify patients with HIV from administrative claims data is a potentially efficient approach to conducting research on the quality of health care received by this patient population, and their health outcomes [1, 2]. In this population-based validation study of women that spanned ICD-9-CM and ICD-10-CM eras, we found that an algorithm requiring ≥ 1 HIV diagnosis code had a PPV of 80.5% (95% CI: 75.4–84.8). The PPV increased appreciably when ≥ 2 HIV diagnosis codes were required (PPV = 93.9% [95% CI: 90.0-96.3]) with little additional increase in the PPV when ≥ 3 HIV diagnosis codes were required (PPV = 97.2 [95% CI: 94.2–98.7]). Similar PPVs were observed for ICD-9-CM and ICD-10-CM HIV diagnosis code-based algorithms. The PPVs of the two most common categories of ICD-9-CM diagnosis codes ranged from 82.7 to 83.7%, and the PPVs of the analogous ICD-10-CM codes ranged from 87.4 to 92.0%. The PPV of the new category of HIV diagnosis codes specific to HIV in pregnancy, childbirth and the puerperium included in ICD-10-CM, was 100% (95% CI: 77.2–100.0), although the confidence interval was wide. Finally, an algorithm that examined the PPV of requiring ≥ 1 HIV diagnosis codes plus ≥ 1 CD4 procedure code on or after the HIV code, was similar to the algorithm that required ≥ 2 HIV diagnosis codes (i.e., 94%).

To our knowledge, few prior studies have validated the use of HIV diagnosis codes alone for identifying patients with HIV, and none have done so for ICD-10-CM codes only. Errors in clinical coding is a well-recognized issue [13] and in the present study we observed a PPV of only 80.5% for an algorithm requiring only ≥ 1 HIV code. During the ICD-9-CM era, Fultz et al. validated the use of HIV diagnosis codes within the US Department of Veterans Affairs Healthcare System (VA) using data from 1998 to 2003 [6]. A PPV of 69% was observed for ≥ 1 HIV diagnosis code, and a PPV of 88% for an algorithm that required ≥ 2 outpatient HIV diagnosis codes or ≥ 1 inpatient HIV diagnosis code. Recently, May et al. validated this second algorithm using 2006–2020 data from UT Physicians, a health care system in the greater Houston area and reported a PPV of 99% (May et al. did not report PPVs separately for diagnosis codes during the ICD-9-CM and ICD-10-CM eras ) [1]. In the present study, we observed a finding similar to May et al. wherein the PPV of ≥ 2 HIV diagnosis codes (outpatient or inpatient) during 2010–2020 was 94% (95% CI: 90.0-96.3). Taken together, these findings suggest that, if the accuracy measure of greatest interest is PPV, as may be the case when the goal is to identify a cohort of patients with HIV in whom health care utilization patterns are to be examined [14], a simple algorithm that requires ≥ 2 HIV diagnosis codes may perform well.

Limitations of our study include that it was conducted at a single health care system; the generalizability of our findings may be impacted if clinicians’ coding practices vary across settings. Further, our study population included only women which may also limit generalizability of our results. However, compared to the May et al. study [1] previously mentioned, we observed a similar PPV for a comparable HIV diagnosis code-based algorithm, using data from a largely overlapping time period, yet their study cohort was comprised of only 36% women. In addition, we were missing race and ethnicity information for 15% of our study population which may limit assessment of the generalizability of our results. Also potentially relevant to generalizability, is that our study population was followed for a mean of 3.1 years following the first HIV diagnosis code during the study period (median 2.0 years [interquartile range: 0.9–3.9]). Further, our study design did not permit estimation of other accuracy measures such as sensitivity, specificity, and negative predictive value [15]. However, we note that the PPV of an algorithm is the accuracy measure of greatest relevance when the goal is to define a cohort of persons with a particular condition (e.g., HIV) [14]. An additional limitation is that the PPV estimates for the less common individual ICD-9-CM and ICD-10-CM HIV diagnosis codes were wide. We also did not distinguish incident from prevalent HIV, thus our results may not be generalizable to identification of only incident or only prevalent HIV. Lastly, our gold standard required a provider’s note or laboratory evidence of HIV infection, and to the degree that this information was missing from the medical charts of patients who had an HIV diagnosis, our PPVs may be underestimates.

Strengths of our study include that we validated the various HIV diagnosis code-based algorithms via medical record review in a population-based sample. The medical records of all women meeting study inclusion criteria were reviewed. We also reported PPVs separately for ICD-10-CM-based algorithms.

## Conclusion

If the accuracy measure of greatest interest is PPV, as may be the case when the goal is to identify a cohort of patients with a particular condition [14], our findings suggest that a simple algorithm using administrative health care data that requires ≥ 2 HIV diagnosis codes may perform well for identifying patients with HIV.

## Data Availability

All data generated or analysed during this study are included in this published article.

## Abbreviations

HIV: Human immunodeficiency virus
KPWA: Kaiser Permanente Washington
ICD-10-CM: International Classification of Diseases Tenth Revision, Clinical Modification
ICD-9-CM: International Classification of Diseases Ninth Revision, Clinical Modification
PROSPR II: Population-based Research to Optimize the Screening Process
SEER: Surveillance Epidemiology and End Results registry
PPV: positive predictive value
CI: confidence interval
